# Maternal and Fetal Complications in Pregnant Women with Neurofibromatosis Type 1: Literature Review and Two Case Reports

**DOI:** 10.3390/jcm14020451

**Published:** 2025-01-12

**Authors:** Ancuta Nastac, Anca Maria Panaitescu, Iulia Huluță, Nicolae Gică, Gabriel-Petre Gorecki, Radu Botezatu, Cristina Violeta Tutunaru, Vlad Mihai Voiculescu, Florina Mihaela Nedelea

**Affiliations:** 1 “Carol Davila” University of Medicine and Pharmacy, 020021 Bucharest, Romania; 2 Department of Obstetrics and Gynecology, Carol Davila University of Medicine and Pharmacy, “Filantropia“ Clinical Hospital, 011171 Bucharest, Romania; 3 Department of Anesthesia and Intensive Care, Faculty of Medicine, “Titu Maiorescu” University, 031593 Bucharest, Romania; 4 Department of Anesthesia and Intensive Care, CF2 Clinical Hospital, 011464 Bucharest, Romania; 5 Department of Dermatology, Faculty of Medicine, University of Medicine and Pharmacy of Craiova, 200349 Craiova, Romania; 6 Clinic of Dermatology, Carol Davila University of Pharmacy and Medicine, “Elias” Emergency University Hospital, 011461 Bucharest, Romania

**Keywords:** neurofibromatosis type 1, complications, pregnancy

## Abstract

Neurofibromatosis is a genetic disorder arising de novo or with an autosomal dominant transmission that typically presents either at birth or in early childhood, manifesting through distinctive clinical features such as multiple café-au-lait spots, benign tumors in the skin, bone enlargement, and deformities. This literature review aims to resume the spectrum of maternal and fetal complications encountered in pregnant women with neurofibromatosis type 1 (NF1). Thorough research was conducted on databases such as Web of Science, PubMed, Science Direct, Google Scholar, and Wiley Online Library. This review includes 48 case reports, original studies, and reviews on NF1 in pregnancy. The research on the interlink between NF1 and fertility and its influence on human-assisted reproduction techniques is limited. Preimplantation testing (by in vitro fertilization) and prenatal diagnosis (by chorionic villus sampling or amniocentesis) are available to detect affected fetuses. However, genotype–phenotype correlation is difficult to predict. Preconceptional planning and targeted investigations are crucial in understanding the extent of maternal disease. Although in some cases lesions can evolve rapidly during pregnancy, most pregnancies and births in NF1 go well with careful planning. There is a higher incidence of pheochromocytomas and pre-eclampsia, vascular rupture, and cardio-respiratory issues. Anesthesia at birth is a challenge in most cases, and before offering spinal anesthesia, imaging tests should be performed to characterize spinal lesions. General anesthesia may also be challenging when the disease affects the face, neck, upper spine, or airways. Birth-related difficulties may arise because of large neurofibromas located at the level of skin incision or birth canal; uterine atony may be expected if there are uterine lesions. Some complications can develop in postpartum, and affected women should be carefully followed even after pregnancy. Fetal risks include preterm birth (spontaneous or iatrogenic), growth restriction and developmental issues, birth complications, cardiovascular risk, and fetal/neonatal demise. Pregnancies in women with NF1 should be regarded as high-risk and followed in a multidisciplinary fashion. Careful assessment of lesions is of utmost importance before and during pregnancy for anticipating potential maternal risks and before birth to plan anesthesia and delivery.

## 1. Introduction

Neurofibromatosis encompasses a spectrum of genetic disorders predisposing to tumor growth, with neurofibromatosis type 1 (NF1) occurring in approximately one in 3500 births and neurofibromatosis type 2 (NF2) and schwannomatosis in roughly one in 40,000 births. These conditions follow an autosomal dominant (AD) inheritance pattern, carrying a 50-percent risk of transmission to offspring from affected parents [1]. However, manifestations in the offspring can range from very mild to extremely severe with limited possibility to predict the genotype–phenotype correlation [2].

NF1, known as von Recklinghausen disease, represents a prevalent AD genetic disorder affecting approximately 1 in 3000 individuals globally [3]. The NF1 gene resides on chromosome 17 at the 17q11.2 locus. NF1 typically presents either at birth or in early childhood, manifesting through distinctive clinical features like the presence of multiple café-au-lait spots, primarily clustered in regions such as the groin and underarms, alongside the development of benign tumors beneath the skin. Additionally, individuals with NF1 may exhibit bone enlargement and deformities, as well as spinal curvature. Notably, there is potential for the emergence of tumors within the central nervous system, affecting areas such as the brain, cranial nerves, or spinal cord [1,4]. Cognitive challenges, potential learning disabilities, and neuroatypical behavior (attention-deficit/hyperactivity disorder, autism spectrum disorders) are considered more prevalent in the NF1 population compared to the general population [5]. There is also an increased risk of vasculopathy, vascular abnormalities in major arteries, and a higher cardiovascular risk in these patients [6].

NF1 represents a challenge for pregnancy as there are various risks for both the expectant mother and the developing fetus, necessitating a comprehensive understanding of potential complications to optimize perinatal counseling and care.

Pregnancy in women with NF1 may bring potential exacerbation of existing symptoms and the onset of novel complications. It is very important to note that the majority of pregnant women with NF1 have uncomplicated pregnancies. However, a subset confronts risks warranting vigilant monitoring and tailored interventions. In this literature review, we aim to resume the spectrum of maternal and fetal complications encountered in pregnant women with NF1.

## 2. Materials and Methods

To write this literature review, meticulous research was conducted using the following scientific databases: Web of Science, PubMed, Science Direct, Google Scholar, and Wiley Online Library, aiming for a synthesis of contemporary literature and recent clinical insights. We selected relevant studies dating from 2010 to 2024, as well as data from the National Institute of Health. A total number of 48 case reports, original studies, and reviews were selected and cited in this paper; we included two of our remarkable cases. When choosing the articles, only the ones portraying pregnant women diagnosed with NF1, newborns of NF1 mothers, or newborns diagnosed with NF1 were taken into consideration. Data related to NF1 in the general female population were not taken into consideration, and this paper does not cover management and therapy strategies for this genetic disorder. Toward the end of the paper, we added two case reports from our clinic to enrich the existing literature.

## 3. Results

### 3.1. NF1 and Fertility

As concluded from our extensive literature search, we can state that there is little to no data regarding the interlink between neurofibromatosis and fertility. However, below, we will present the results from two interesting studies conducted on human-assisted reproduction candidates diagnosed with NF1.

### 3.2. NF1 and In Vitro Fertilization (IVF)

NF1 complicates reproductive decision-making, and one of the options to prevent the birth of an affected offspring is preimplantation genetic testing (PGT). A retrospective study conducted at the Dutch PGT expert center on data obtained from 23 years of clinical experience analyzed 82 couples affected by NF1 who chose to have PGT [7]. Fertility assessments revealed a higher percentage of male infertility in males with NF1 compared to partners of affected females. Cardiac evaluations in women with NF1 showed no contraindications for IVF or pregnancy. In another study that provided insights regarding this topic, the percentage of embryos with NF1 mutation was calculated [8]. There were a total of 77 couples, and from 80% of biopsied embryos, 46% were unaffected by the parental NF1 mutation. Live birth was confirmed in 14% of cycles, with an expected 21% live birth rate. It is a domain worth exploring, but for the moment, these were the only two studies found in the existing literature. Early counseling on fertility and reproductive options using disease-specific data is essential to set appropriate expectations. These findings provide valuable insights into the success rate of PGT for NF1, underscoring its importance.

### 3.3. Preconceptional Planning and Counseling

Detection of mutations in the NF1 gene allows either for PGT or for prenatal diagnosis, but it is complex and time-consuming due to the gene’s large size, the existence of pseudogenes, the lack of mutation clustering, and the variability of clinical findings. Given the restricted time for investigations in prenatal diagnosis, detecting disease-associated NF1 alleles is more rapid and useful, especially in familial cases. The phenotypic variability and lack of genotype–phenotype correlation complicate reproductive decisions for NF1 families, emphasizing the need for appropriate and thorough discussions of potential outcomes before fetal genetic testing.

A five-year study on prenatal diagnosis (PND) for neurofibromatosis type 1 (NF1) involving 146 women who underwent 205 procedures, primarily chorionic villus biopsies (88%), found the NF1 variant in 85 fetuses (41%), while 122 tested negative (59%). Out of 207 fetuses, 135 pregnancies were carried to term, resulting in 119 unaffected and 16 affected by NF1. Additionally, 69 pregnancies were terminated due to an affected fetus, with two miscarriages and one in utero death reported. Notably, two cases of germline mosaicism were identified, where mothers with mosaic NF1 had negative test results for their fetuses, revealing limitations in indirect PND methods. These findings highlight the importance of accurate genetic counseling and the evolving landscape of PND techniques, which present both ethical challenges and opportunities for non-invasive testing [9].

Preconception counseling is crucial and superior to post-conception discovery and management, as it allows for better anticipation of future medical issues. It should also focus on evaluating the extension of NF1 disease in the future mother since this seems to affect pregnancy outcomes. Genetic counseling and screening should be initiated before conception. Couples should be guided through the decision-making process, including the option of terminating the pregnancy if significant risks are identified. This comprehensive approach ensures that affected women are well informed and supported throughout their reproductive planning. As a general rule, pregnant women should begin taking folic acid before conception to reduce the risk of neural tube defects in their children, regardless of the presence of NF1 [10,11].

### 3.4. NF1 Effects on Pregnancy

#### 3.4.1. Pregnancy in Women with NF1

Despite advancements in understanding NF1 during pregnancy, it remains consistent that pregnant women with this condition experience an increased risk of obstetrical complications. In a retrospective register-based populational study conducted in Finland, it was stated that for mothers with NF1, pregnancies are shorter, and cesarean deliveries are more frequent and necessary. It is mentioned that common complications among pregnant women with NF1 include pre-eclampsia, poor fetal growth, oligohydramnios, and placental abruption. Notably, this study provides the first evidence that fetuses affected by NF1 determine shorter gestational durations [12].

There are recently reported cases where this condition manifested its initial signs during pregnancy. For example, eruptive neurofibromas were reported in one case report [13] as being the initial manifestation of NF1, emerging three months after a positive pregnancy test. Typically, however, patients are diagnosed with NF1 at an early stage of life, and genetic counseling is sought before pregnancy planning. Nevertheless, if the diagnosis is not established and individuals become pregnant, although there are increased risks, the evolution of the pregnancy can still proceed normally [14,15]. It is worth mentioning a case report from 2021, which describes a woman diagnosed with NF1 and human immunodeficiency virus (HIV), who, with appropriate counseling and under careful medical supervision, gave birth to four HIV-free newborns with no complications associated with NF1 [16]. PND to determine the NF1 mutation in the fetus in the context of maternal HIV presents a potential risk of maternal–fetal transmission, complicating counseling and decision-making for women [17].

A study conducted in 2020 on the growth dynamics of neurofibromas surprises with findings that counteract the already-published data. It proves that in pregnant NF1 patients, plexiform and cutaneous neurofibroma growth does not differ notably from the general NF1 population, and remarkably, NF1-related symptoms do not show significant changes throughout pregnancy. There was indeed a notable increase in the growth of cutaneous neurofibromas over time, regardless of pregnancy status [18]. Moreover, another study conducted on the Danish population discloses a possible increased risk of stillbirth or spontaneous abortion in NF1 pregnant patients [19]. However, after analyzing the latest findings, it is still not clear from the literature whether pregnancy worsens the long-term evolution of NF1 [18].

Table 1 presents a summary of maternal complications described in the case reports included in this review.

#### 3.4.2. Fetal Outcomes

It is of great importance to focus on fetal outcomes, too. A Finnish study revealed novel findings indicating that anomalies in the urinary system and those pertaining to the eye, ear, face, and neck occur more frequently among children with NF1.

Furthermore, NF1 children exhibited a significantly higher risk of congenital anomalies affecting the circulatory and musculoskeletal systems. Interestingly, non-NF1 children of mothers with NF1 did not exhibit a higher prevalence of anomalies compared to children from non-NF1 mothers [20].

Moreover, Tettamanti et al. [21] mentioned some birth characteristics that may be found in NF1 newborns. These infants displayed distinctive features such as short length, large head circumference, and low Apgar scores. It was also implied that these children are more likely to have high birth weights.

In the Table 2, we will make a summary of fetal outcomes found in the case reports we described.

### 3.5. Genetics

NF1 is caused by heterozygous loss-of-function mutations in the neurofibrin gene on chromosome 17q11.2, with more than 95% of patients meeting the National Institute of Health diagnostic criteria showing such mutations. Because NF1 is a large gene, more than 3000 mutations have been published. The disease exhibits almost 100% penetrance in adults but shows significant variability within and between families, and there is a poor correlation between genotype and phenotype.

The genetic testing approach may be different and requires multidisciplinary evaluation and pretesting genetic counseling. Therefore, for familial cases or when at least two or more of the clinical features are stated for NF1, single-gene testing of the NF1 gene may be considered. Because about 30% of the pathogenic variants affect splicing, and those cannot be detected by the sequencing of genomic DNA(gDNA), additional sequencing of complementary DNA (cDNA, copied from mRNA) increases detection rates [22].

Because 11–13% of the underlying cause may be represented by large deletions/duplications (including NF1 gene) that may not be detected by the sequence analysis of the coding and flanking intronic regions of genomic DNA, other tests may be necessary, such as quantitative PCR, multiplex ligation-dependent probe amplification (MLPA), and chromosomal microarray (CMA) that includes the NF1 gene and/or 17q11.2 chromosomal region.

If the clinical phenotype is not typical, either multi-gene panels (that include NF1, SPRED1 genes, or rasopathy multigene panel) or comprehensive genomic testing (as exome sequencing or genome sequencing) may be considered [23,24].

Table 3 presents a summary of the case reports on NF1 in pregnancy published in the last 10 years. It aims to highlight the main findings regarding possible maternal and fetal complications.

### 3.6. Anesthesia and Delivery in Patients with NF1

Anesthesia during labor and delivery may pose significant challenges in patients with NF1. Cerebral and spinal neurofibromas are common, and there may also be cerebrovascular disease involved, making spinal anesthesia risky in patients with NF1. Imaging studies like MRI late in pregnancy best describe the extension of lesions and help in guiding decisions. On the other hand, GA may not come at ease—in some patients with NF1, there may be airway involvement with neurofibroma of the tongue, larynx, or pharynx that may interfere with tracheal intubation. A history of dysarthria, change of voice, stridor, or dysphagia can suggest airway involvement. Spine deformities (scoliosis/kyphosis) or intrapulmonary lesions may further impede the feasibility of GA. Moreover, there are cardiovascular challenges to keep in mind in these patients—hypertensive disease, vascular fragility, and hypertrophic cardiac disease [47,48].

In the section below, we will provide a full description of the last two cases we mentioned in Table 3.

## 4. Case Reports

### 4.1. Case 1

**Figure 1:**
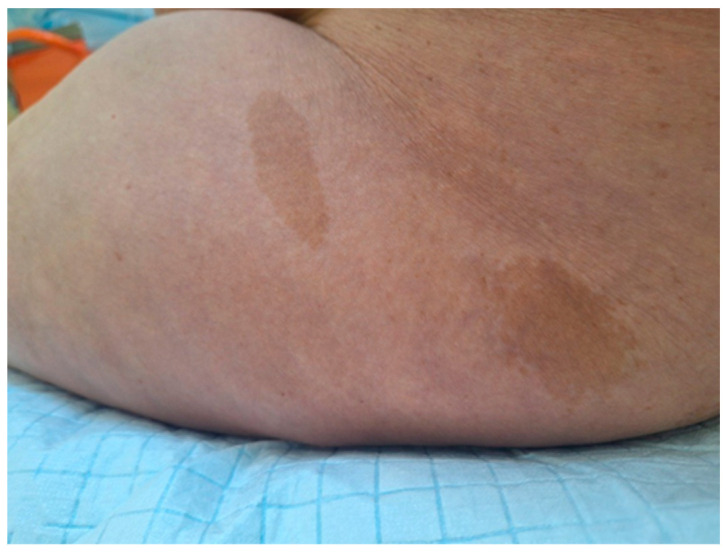
Patient’s eruption café-au-lait spots.

A 24-year-old primigravida with a known history of NF1, with charactersitic café-au-lait spots (Figure 1) but with no adequate prenatal care presented at 40 weeks gestation in active labor. She was offered nitrous oxide inhaled anesthetic gas for pain management during labor since there were no imagistic studies to guide anesthesia decisions. She delivered a male infant weighing 3250 g AI 7/8 via vacuum-assisted vaginal delivery (KIWI ventouse). He had an AI of 7 at 1 min and of 8 at 5 min. Recovery was uneventful, but the woman and child were lost to follow-up. The case highlights the difficulties in anesthesia counseling in the context of inadequate prenatal care).

### 4.2. Case 2

**Figure 2:**
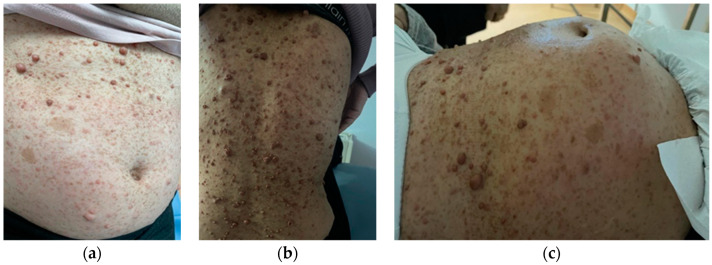
(**a**–**c**) Patient no. 2′s eruption (multiple café-au-lait spots and brown soft tumors disseminated on the patient’s skin).

A 33-year-old primigravida with a history of NF1 and adequate prenatal care entered spontaneous labor at 37 weeks of gestation. She was planned for a CS with GA at 38 weeks. During her pregnancy, the neurocutaneous lesions grew and disseminated (Figure 2), and she was also proven to have uterine neurofibromas on prenatal ultrasound scans. She was counseled about the risk of preterm delivery and postpartum hemorrhage in the context of uterine involvement. She also developed hypertension controlled with oral anti-hypertensive medication in the last few weeks of pregnancy. She delivered by emergency CS under GA a male infant weighing 2200 g with AI of 8/9. Intrapartum hemorrhage was experienced due to abnormal uterine retraction but responded well to typical medical management (carbetocin, misoprostol). The postpartum course was uneventful. The case highlights the risk of postpartum bleeding when uterine neurofibromas are present.

## 5. Conclusions

NF1 is a genetic disorder that requires continuous medical attention and a multidisciplinary approach to manage its various effects. Characterized by the development of neurofibromas, this condition can have serious health implications. In this paper, we have navigated the effects of NF1 in pregnant patients and its potential complications that significantly impact both maternal and fetal health during pregnancy and postpartum. One of the primary concerns in pregnant women with NF1 is the dangerous growth of the neurofibromas, which can pose significant health risks. In addition, other serious complications associated with NF1 during pregnancy include pheochromocytoma, which can cause severe hypertension and placental abruption, leading to potential fetal distress or stillbirth. Moreover, rare but severe complications, like haemothorax and haemoperitoneum from ruptured vessels, can occur. These conditions require immediate medical attention and can be life-threatening for both the mother and the fetus. Given the potential for such severe complications, the importance of regular screening and careful monitoring throughout pregnancy should not be neglected. Early detection and intervention are key to managing NF1 during pregnancy, allowing for timely medical or surgical interventions if necessary and ensuring the health and safety of both the mother and her unborn child. It is of great importance that the medical team does thorough prenatal counseling and ensures that the patient is aware of all the possible risks and complications that can occur. Further research is necessary in the field of NF1 in pregnancy to improve current knowledge and generate applicable general medical recommendations.

## Figures and Tables

**Table 1:** Maternal complications reported in pregnancies with NF1.

**Neurofibromas**: transformation of pre-existing neurofibromas; glial tumor with malignant degeneration; multiplication of eruptive neurofibromas; growth and enlargement of pre-existing neurofibromas
**Vascular complications**: aortic rupture; spontaneous massive hemothorax due to ruptured intercostal artery
**Respiratory issues**: restrictive respiratory dysfunction; pulmonary edema; pleural effusion
**Cardiac issues:** mitral and tricuspid regurgitation; restrictive cardiac insufficiency
**Pain and neuropathic complications**: cauda equina syndrome
**Hypertensive disorders**: pre-eclampsia/eclampsia; hypertension; pheochromocytoma
**Placental complications**: placental abruption; placenta previa
**Postpartum complications**: death due to the rapid progression of neurofibromas postpartum, causing local destruction, or due to vascular ruptures

**Table 2:** Fetal complications reported in pregnancies with NF1.

**Growth and development issues**: intrauterine growth restriction; congenital deformities of the feet
**Birth complications**: preterm birth; breech presentation
**Cardiovascular issues**: fetal tachycardia
**Fetal demise**/**neonatal death**

**Table 3:** Summary of maternal and fetal complications in case reports from the last 10 years.

Article	NF1 Known Before Pregnancy/Co-Morbidities	Birth	Maternal Complications	Fetal/Neonatal Outcomes	Management
Zúñiga et al., 2015 [25]	Yes	Elective CS, 38 wks; GA	Transformation of a nodular, small neurofibroma to a cystic, large neurofibroma in the cervical region accompanied by mass effect: dysphonia, dysphagia, local pain (during pregnancy)	BW—2800 g, AI 1 min—8; no follow-up for NF1	Surgical approach—resection of the neurofibroma after birth with no further complications
Zafer et al., 2015 [26]	No	Emergency CS, 30 wks; neuraxial anesthesia	Pre-eclampsia/ eclampsia Glial tumor with malignant degeneration—growth of a neurofibroma (during pregnancy)	BW—2345 g, AI at 1 min—5, at 5 min—8; no follow-up for NF1	Magnesium sulfate neuroprophylaxis, MRI imaging for follow-up and evolution
Remón-Ruiz et al., 2017 [27]	Yes	Emergency CS for placental abruption at 35 wks	Pheochromocytoma (diagnosed during 3rd pregnancy) Placental abruption History: 1st pregnancy—stillbirth, 2nd pregnancy—abruption	BW—1610 g, no other data	Laparoscopic, converted to open adrenalectomy at 23 wks of pregnancy
Galvan et al., 2018 [28]	Yes; co-morbidities SLE with past serositis	IOL at 31 wks for PE; combined spinal-epidural catheter, after MRI exclusion of spinal lesions; vaginal delivery	Pre-eclampsia	No fetal or neonatal data	Hypertension management, induction of labor for PE with MRI during cervical ripening to rule out any spinal neurofibromas
Tateishi et al., 2018 [29]	Yes; previous hypertension, subarachnoid hemorrhage 15 yrs before	Emergency CS, peripartum hysterectomy at 30 wks due to ascending aorta rupture	Ascending aortic rupture at 30 wks; pleural effusion	BW—1482 g; recovered well, discharged day 67	Chest CT; peripartum hysterectomy, cardiopulmonary bypass; 8 mm small aortic tear with rupture of the ascending aorta was repaired; complete recovery
Williams et al., 2018 [30]	Yes	No reported data	Enlarging of a plexiform tumor on the thigh (during pregnancy)	No reported data	Biopsies for diagnosis and to rule out malignancy
Kalamantis et al., 2018 [31]	Yes	Emergency CS for preterm labor at 36 wks, GA, previous MRI with lesions	Placenta previa	BW—2650 g, normal outcome; not affected by NF1	Offered prenatal testing for NF1 by amniocentesis; declined testing
Silva et al., 2019 [32]	Yes	Vaginal delivery at full term; peridural contraindicated; local anesthesia and intravenous remifentanil	Neuropathic pain due to T12–L2 meningocele, with cauda equina distortion; cessation of opioids (during pregnancy)	-	CVS at 11 wks excluded NF1 in the fetus; transcutaneous electrical nerve stimulation for pain management throughout pregnancy
Sathiamurthy et al., 2019 [33]	No	Term delivery; no other data	Spontaneous massive hemothorax due to a ruptured intercostal arterio-venous malformation on the 4th day postpartum in the context of NF1	No data	Emergency thoracotomy, hematoma extending into spinal canal, laminectomy; paraplegic with loss of bladder and sphincter functions Histopathology result revealed mesenchymal proliferative lesions, consistent with plexiform neurofibroma
Kermaj et al., 2019 [34]	Yes	Planned CS at 38 wks	Pheochromocytoma diagnosed at 33 wks	Normal outcome, no other data	MRI, α/β blockers, aspirin, doxazosine to control BP
Sánchez-Contreras et al., 2020 [35]	Yes	CS, no other data	Massive spontaneous hemoperitoneum postpartum day 5	No data	Repeated laparotomy, eventual death, autopsy—arterial dissection, due to NF1
Yahya et al., 2020 [36]	No	1st pregnancy uneventful, 2nd pregnancy uneventful	Multiplication of eruptive neurofibromas (during both pregnancies)	No data	Clinical diagnosis of NF1 and histology positive for neurofibroma
Jasim et al., 2020 [37]	Yes	Vaginal delivery at 35 wks	Pheochromocytoma misdiagnosed as pre-eclampsia	No data	Adrenalectomy postpartum
LaBan et al., 2021 [38]	Yes	No data	Increase in size and number of pedunculated NF skin lesions, cauda equina syndrome during pregnancy	No data	No data
Bansal et al., 2021 [39]	Yes	Emergency CS, 28 wks for PE and FGR, spinal anesthesia	Hypertension, pulmonary edema, mitral and tricuspid regurgitation, pleural effusion, mild cardiomegaly, multiple neurofibromas	BW—686 g; discharged well at 1500 g; no follow-up for NF1	Difficulties in incision due to NF1 lesions
Hashimoto et al., 2021 [40]	Yes	Emergency CS at 34 wks for abnormal CTG trace and maternal instability, GA	Sudden pleuritic pain and SOB; Hemothorax due to rupture of the intercostal artery invaded by neurofibromas	Fetal demise, neonatal death	Endovascular coil embolization after CS
Zeitler et al., 2022 [41]	Yes	Emergency CS at 33 wks;	Pheochromocytoma with malignant transformation during pregnancy	-	ECMO
Cîrstoiu et al., 2022 [42]	Yes (NF1 and Becker nevus syndrome)	Emergency CS	Severe scoliosis, restrictive respiratory dysfunction, restrictive cardiac insufficiency, Becker nevus, neurofibromas, cachectic state	Preterm birth, fetal tachycardia	Emergency C-section, postpartum biopsy, respiratory support
Odongo et al., 2022 [43]	No	Iterative CS, 40 wks	Death due to rapid progression of a giant cutaneous plexiform neurofibroma with local destruction, 11 wks postpartum	Normal outcome, NF1 status not known	Skin biopsies, no histological findings of malignancy
Kitamoto et.al, 2022 [44]	Yes	Pregnancy 1: Emergency CS at 34 wks	Pleuritic pain, SOB; rupture of the left intercostal artery due to NF1 vascular fragility causing hemothorax	Neonatal death	Embolization; recovered well
		Pregnancy 2–3 moths after CS; planned CS at 31 wks; GA	Hospitalized from 26 wks	BW—1338 g, AI 1/3	3 months postpartum 8th intercostal artery rupture, maternal death
Ibishi et al., 2023 [45]	Yes	Emergency CS	Growth of pre-existing neurofibromas during pregnancy Placental abruption	BW—2760 g, AI—5/6, recovered well, no NF1	
Olumodeji et al., 2024 [46]	No	Planned CS at 38 wks for breech	Diagnosed at delivery	Breech presentation, genus varus and bilateral talipes equinovarus; no other complications	
**Case 1**	Yes	40 wks, primipara, spontaneous labor; vaginal delivery	Nitric oxide for pain management during labor	Normal outcome, not known NF1 status in offspring	Counseled for anesthesia options
**Case 2**	Yes	37 wks, ruptured membranes, PE, CS under GA	Postpartum hemorrhage due to uterine lesions	BW—2200 g, AI 8/9; no NF1	Medical management of postpartum hemorrhage

CS—Cesarean section; GA—general anesthesia; wks—weeks; g.—grams; AI—Apgar index; BW—birth weight; IOL—induction of labor; PE—pre-eclampsia; SLE—systemic lupus erythematosus; yrs—years; CVS—chorionic villus sampling; BP—blood pressure; FGR—fetal growth restriction; CTG—cardiotocography; SOB—shortness of breath; ECMO—extracorporeal membrane oxygenation.
